# Modifications of the HJC (Holmquist–Johnson–Cook) Model for an Improved Numerical Simulation of Roller Compacted Concrete (RCC) Structures Subjected to Impact Loadings

**DOI:** 10.3390/ma13061361

**Published:** 2020-03-17

**Authors:** Chao Wang, Ran Song, Gaohui Wang, Sherong Zhang, Xuexing Cao, Peiyong Wei

**Affiliations:** 1 State Key Laboratory of Water Resources and Hydropower Engineering Science, Wuhan University, Wuhan 430072, China; janson126@163.com (C.W.); wanggaohui@whu.edu.cn (G.W.); 2 State Key Laboratory of Hydraulic Engineering Simulation and Safety, Tianjin University, Tianjin 300350, China; sdlwwpy@outlook.com; 3 School of Civil Engineering, Tianjin University, Tianjin 300350, China; 4 The Third Railway Survey and Design Institute Group Corporation, Tianjin 300142, China; tjudam@126.com; 5 Huaneng Lancang River Hydropower INC., Kunming 650051, China

**Keywords:** roller compacted concrete, modified HJC model, dynamic compressive properties, SHPB experiment, numerical predictions

## Abstract

Structures made of Roller Compacted Concrete (RCC) may be subjected to dynamic loads during their service life. Understanding the dynamic material properties of RCC and the performance of RCC structures is essential for better analysis and design of RCC structures. As full-scale tests are often unaffordable, numerical simulation methods are continuously employed. However, in numerical simulations, determining a reasonable constitutive relationship for RCC materials is still limited due to the complexity of the composite and the special rolling and compacting construction technology. In this paper, the triaxial compressive test and split Hopkinson pressure bar (SHPB) experimental results for RCC are introduced as an experimental foundation. Parameter calibrations and modifications in terms of the strength yield surface, the strain rate effect and the failure criterion for the RCC materials are presented. Numerical verification is illustrated for simulating the SHPB experiment and predicting the dynamic compressive characteristics of RCC specimens with a modified HJC model. The results reveal that the simulation results for the modified model have better agreement with the test data than those with the model before modification and have better simulation results. Sensitivity studies of the key parameters on the yield surface of the modified HJC model are conducted to improve the simulation effect for numerically predicting the performance of RCC structures exposed to explosive and impact loads.

## 1. Introduction

Roller compacted concrete (RCC) material has been widely applied in the construction of infrastructure such as hydraulic structures since the early 1970s due to its advantages of cost-effectiveness and rapid construction [1,2,3]. Recently, a series of 100–300 m world-class high RCC dams have been under construction or built. During construction, the RCC mixture is paved and spread using a bulldozer and then compacted to a layered structure by using a vibratory roller. RCC, as a special type of concrete material, has a different mixture from traditional concrete, for example, using less water and more fly ash to replace Portland cement [1]. During their service life, these structures may be subjected to different types of loads, such as blast and impact loads. Therefore, it is necessary to understand the dynamic mechanical properties of RCC materials as well as the dynamic performance of the RCC dams. 

Predicting the performance of RCC structures subjected to explosive and impact loads through full-scale tests is often beyond affordability. Numerical simulation has proven to be an effective method for understanding the dynamic performance of RCC dams subjected to blast and impact loads. In a numerical simulation, a sound constitutive model selected to represent the dynamic mechanical properties of RCC under a high strain rate is vital to ensure the validity of the numerical simulation [4]. Numerical analyses have shown that the dynamic constitutive relationship of the material and the compressive strength, ultimate deformation and elastic modulus can greatly influence the accurate modelling of the structural dynamic response [5,6]. For describing the dynamic mechanical behaviour of concrete-like materials, several comprehensive constitutive models have been studied with the consideration of the effect of hydrostatic pressure, strain rate strengthening and strain softening, such as the Holmquist–Johnson–Cook (HJC) model [7], the Riedel–Hiermaier–Thoma (RHT) model [8], the Taylor–Chen–Kuszmaul (TCK) model [9], the continuous smooth cap (CSC) model [10] and the Karagozian & Case (K&C) model [11]. Among these, the K&C model, RHT model and HJC model are established based on the plasticity and damage theories in DYNA3D software [12], while the CSC model is based on visco-plastic and damage theories considering the coupled volumetric and shear behaviour [13]. The K&C model and RHT model use three yield surfaces in terms of the initial elastic yield surface, the failure surface and the residual surface to describe the strength property. All of these dynamic constitutive models refer to many parameters for describing the complicated mechanical behaviour, making it difficult to determine a suitable and effective model for RCC. The HJC model considers the strength yield surface, the damage accumulation and the strain rate effect, which contains fewer parameters to be determined, and the model can present the dynamic compressive behaviour of concrete well.

Though the parameters of the original HJC model can be obtained from the literature [14], the automatically generated parameters cannot avoid errors when the original HJC model is used to evaluate the blast resistance of RCC structures subjected to underwater explosion because the material behaviour of RCC deviates from the regular behaviour of normal concrete. For example, the properties and stress–strain model are both different for RCC from normal concrete [15,16]. The composition of the materials and the difference in the stress state of the structure would cause a significant distinction in the meso-stress field, deformation and failure forms [4]. For the brittleness of RCC, many factors may affect the validity and accuracy of the experimental results [5]. Appropriate parameters or a sound dynamic constitutive model for RCC are almost impossible to find in existing studies, and parameter calibrations of the HJC model for RCC materials are important based on the experimental observations and unique characteristics of RCC materials.

To this end, sound constitutive models are still lacking that can be used to describe the dynamic properties of RCC materials and predict the dynamic behaviour of RCC structures. The present study aims to provide an appropriate constitutive relationship for RCC under a dynamic compressive state. In this paper, modifications are made for the parameters of the strength surfaces and the dynamic increase factor for compression. A modified HJC material model representing the dynamic properties of RCC under a high strain rate and its corresponding parameters are established and validated for the improvement of a numerical simulation of the dynamic response of RCC compared to the test data. Furthermore, sensitivity analyses of the parameters that control the yield surface are carried out to understand the influence of each individual parameter.

## 2. Experimental Observations for RCC Materials

The experimental observations before the modifications to the HJC model for RCC are briefly introduced in this section, combining the uniaxial compressive test results, the triaxial compressive test results and the dynamic compressive test results.

### 2.1. Uniaxial and Triaxial Compressive Results

Uniaxial and triaxial compressive data for RCC materials are crucial to define the failure surface in the modified HJC model for RCC materials. The RCC mixture in this study is made of the mortar matrix, aggregates and additive. The mortar matrix is a mixture of water, cement, sand, fly ash, a water reducing agent and an air-entraining agent. The water-cement ratio (W/C) is set to 0.50. The fly ash content and the sand ratio are 60% and 31% by weight, respectively. The details of the RCC mixture and the preparation process of the specimens are given in our previous studies [17,18,19,20], and details of the RCC mixture are given in Table 1.

Uniaxial and triaxial compression tests were performed at the State Key Laboratory of Hydraulic Engineering Simulation and Safety, Tianjin University. The uniaxial compressive tests on the ∅100–200 mm specimens were conducted using an electro-hydraulic servo-controlled loading test machine. The testing machine delivers a constant crosshead movement with a loading rate of 1.20 mm/min, corresponding to a quasi-static strain rate of 1 × 10^−4^/s. The 90-day uniaxial compressive strength $f_{c}^{\prime }$, Young’s modulus and critical strain of the RCC were measured as 20.86 MPa, 2.68 GPa and 0.76%, respectively. 

The triaxial compressive tests were also conducted using the ∅100–200 mm specimens. The confining pressure of the cell can be up to 100 MPa, while the loading mode can be switched between displacement control and load control. The confining pressure $\sigma_{3}$ can be directly measured by a pressure transducer inside the intensifier. The axial load $F_{d}$ is applied by an axial actuator and measured by an in-vessel load cell. The actual axial stress $\sigma_{1}$ can be expressed by Equation (1):

(1) $$\sigma_{1}=F_{d}/A_{s}+\sigma_{3}$$

where $A_{s}$ is the cross-sectional area of the specimens.

An axial extensometer and circumferential extensometer were employed to measure axial displacement and circumferential deformation, respectively. In this way, the total circumferential deformation along the whole perimeter of the cylinder was obtained, which was utilized to derive the average lateral strain $\varepsilon_{3}$. Under uniaxial compression, a displacement control mode was used to obtain the stress–strain curve, with a rate of 0.002 mm/s. The confining pressure was first applied to the target value in load control mode and then kept constant. After the target $\sigma_{3}$ was obtained, the control mode was switched to displacement control and deviatoric stress was applied until the fracture of the RCC specimens occurred, with a rate of 0.002 mm/s.

**Figure 1:**
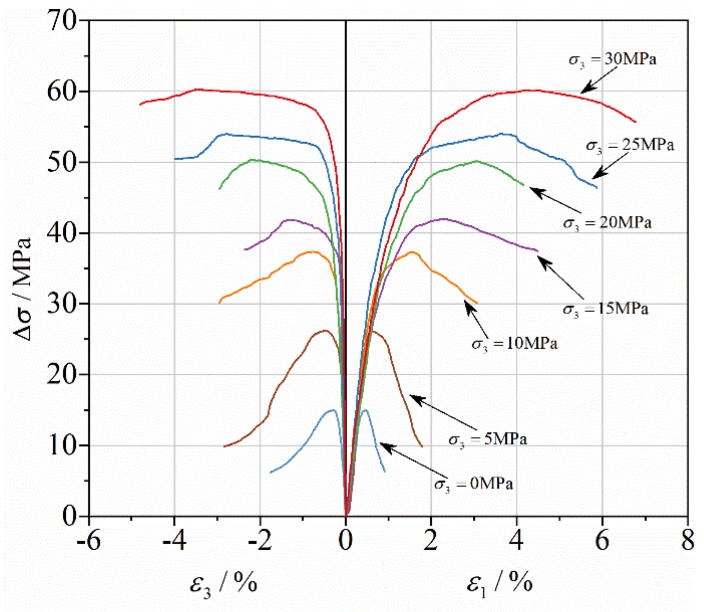
Axial deviatoric stress versus strain curves for RCC samples under confining pressures.

A total of seven levels of confining pressure ($\sigma_{3}$) were employed to cover the response under both moderate and high confinement, 0, 5, 10, 15, 20, 25 and 30 MPa, corresponding to confinement ratios of approximately 0.00, 0.34, 0.67, 1.01, 1.34, 1.68 and 2.01, respectively. For each confinement level, at least three reasonable test results were obtained. The test results are shown in Table 2, including the peak additional axial stress ($\Delta \sigma_{p}$), the peak axial strain ($\varepsilon_{1p}$) and lateral strain ($\varepsilon_{3p}$). These results are from the average of the specimens. Figure 1 shows the axial deviatoric stress versus the strain curves for RCC samples under confining pressures.

Equation (2) shows the relationship between the normalized peak axial strain ratio ($\varepsilon_{1p}/\varepsilon_{cp}$) and the confinement ratio ($\sigma_{3}/f_{c}^{\prime }$), in which $\varepsilon_{cp}$ denotes the peak axial strain of RCC under uniaxial compression. A linear relationship between $\varepsilon_{1p}/\varepsilon_{cp}$ and $\sigma_{3}/f_{c}^{\prime }$ with a correlation coefficient of 0.994 ($R^{2}=0.994$) can be expressed as follows:

(2) $$\frac{\varepsilon_{1p}}{\varepsilon_{cp}}=1+4.74\frac{\sigma_{3}}{f_{c}^{\prime }}$$

### 2.2. Split Hopkinson Pressure Bar (SHPB) Experimental Results

**Figure 2:**
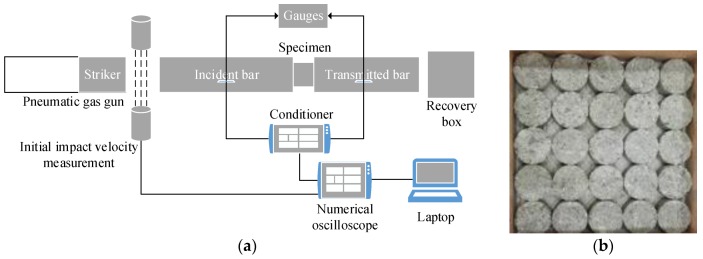
Split Hopkinson pressure bar (SHPB) test system (**a**) schematic diagram of SHPB test system, (**b**) specimen.

**Figure 3:**
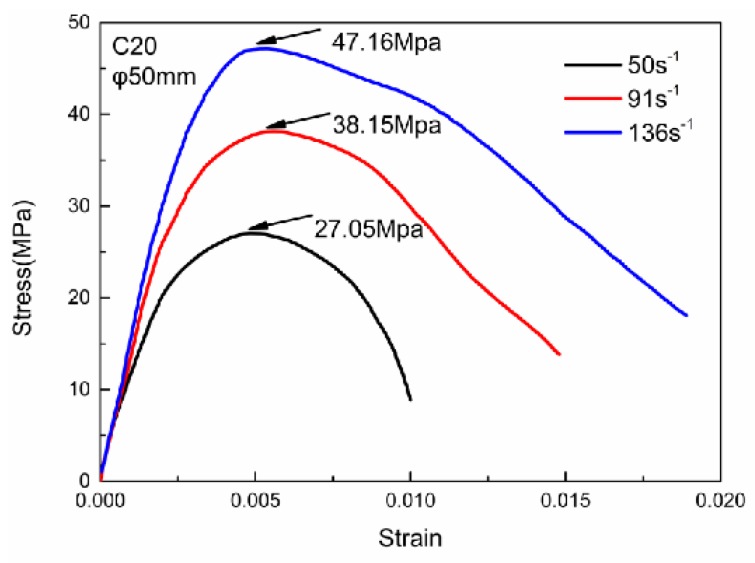
Typical stress–strain curves of RCC specimens.

The ∅50–25 mm specimens were prepared for dynamic compressive tests. The SHPB test system shows in Figure 2. In the SHPB test, the speed of the custom-shaped punch and the input wave strain rate of the incident bar are controlled by adjusting the gas chamber pressure. Two assumptions needed to be satisfied in the SHPB experiment [21]: (1) The stress wave in the pressure bar is in a one-dimensional state. (2) The stress is uniform throughout the specimen. For the ∅50–25 mm specimens, the maximum size of the coarse aggregate is 10 mm to satisfy the requirement of sample size for SHPB test, the medium aggregate size is 5–10 mm, the small aggregate size is 5 mm, and the composition (%) by weight is 40:30:30. Figure 3 plots some typical stress–strain curves of RCC specimens subjected to various strain rates. The compressive strengths of the specimens are 38.15 MPa and 47.16 MPa under average strain rates of 91 s^−1^ and 136 s^−^^1^, which are 54% and 126% higher than the quasi-static uniaxial compressive strength of 20.86 MPa. The peak stress and initial elastic modulus increase with increasing strain rate, which means that RCC material is also strain rate sensitive, as it consists of strain rate effects associated with the changing damage modes and viscosity effects of confined air and water in the pores and exhibits structural effects owing to the lateral inertia confinement, which is similar to the dynamic behaviour of conventional concrete in the literature [22,23]. However, the RCC material exhibits a more obvious plasticity plateau at a higher strain rate, which indicates that RCC material exhibits a dynamic strengthening effect and better ductility with increasing strain rate. 

## 3. The Modified HJC Model

The HJC model is often used for describing the characteristics of normal concrete, although there is a difference between normal concrete and RCC in preparation technology, considering that various indicators are close for these two kinds of concrete and their consistent use in engineering; the modified HJC model is established based on the original model [7], combining new experimental observations for RCC materials in Section 2. From the description above, the strength yield surface is determined by the triaxial compressive experimental data, and the strain rate effect should be considered, which can be obtained from the SHPB experiment of RCC materials.

### 3.1. The Original HJC Model

**Figure 4:**
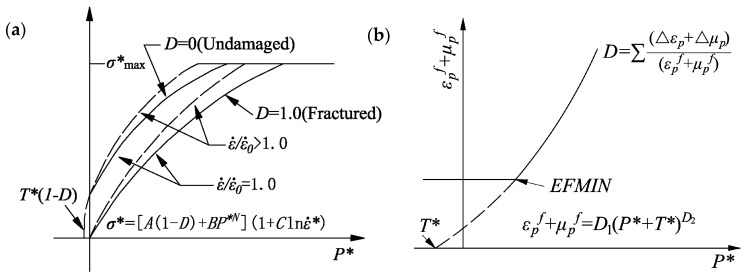

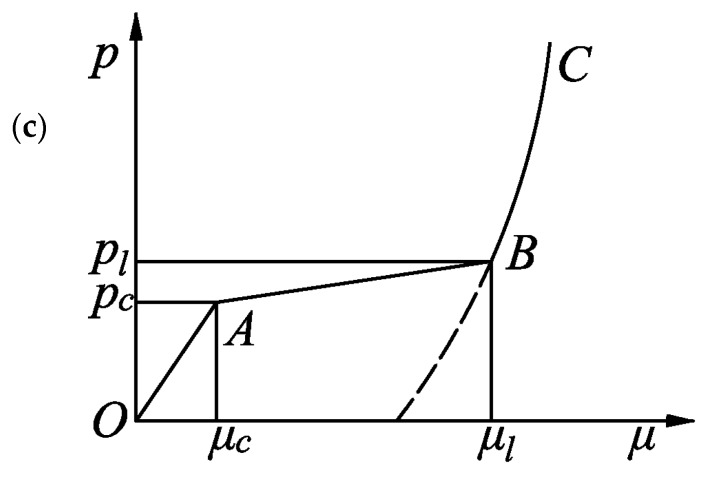
Original Holmquist–Johnson–Cook (HJC) model. (**a**) Equation of the yield surface. (**b**) Damage model of concrete. (**c**) Hydrostatic pressure and volumetric strain curve of concrete.

The original HJC model is introduced briefly before the parameter modifications. Holmquist et al. [24] proposed the HJC model, which considers the influence on yield stress by the damage of the material, strain rate effect and hydrostatic pressure and can effectively describe the compressive behaviour of concrete-like materials under medium and high strain rates. Figure 4 shows the equation of the yield surface, damage model, hydrostatic pressure and volumetric strain curve of concrete.

#### 3.1.1. Yield Function

The yield surface, shown in Figure 4a, of the HJC model can be expressed as follows:

(3) $$\sigma *=\left[A\left(1-D\right)+BP{*}^{N}\right]\left(1+C\ln\dot{\varepsilon }*\right)$$

where $\sigma *=\sigma /f_{c}{}^{\prime }$ is the normalized equivalent stress, σ is the realistic equivalent stress ($\sigma *\leq \sigma {*}_{\max}$, $\sigma {*}_{\max}$ is the maximum normalized strength of concrete), $f_{c}{}^{\prime }$ is the quasi-static uniaxial compressive strength, D is the damage degree (0≤D≤1.0). $P*=p/f_{c}{}^{\prime }$ is the normalized hydrostatic pressure (p is the realistic hydrostatic pressure, shown in Figure 4c). $\dot{\varepsilon }*=\dot{\varepsilon }/{\dot{\varepsilon }}_{0}$ is the dimensionless strain rate ($\dot{\varepsilon }$ is the realistic strain rate, and ${\dot{\varepsilon }}_{0}$ is the reference strain rate). A, B, N and C are constants, where A is the normalized cohesive strength, B is the normalized pressure hardening, N is the pressure hardening exponent and C is the strain rate coefficient.

The strain rate effect is included in Equation (3), and the determination of C is important to describe the strain rate effect in the constitutive model [25]. This relationship will be fitted according to the SHPB experimental data of RCC materials.

#### 3.1.2. Damage Evolution

The damage is described by the equivalent plastic strain and plastic volumetric strain for the HJC model, and the damage evolution equation is

(4) $$D=\sum \frac{\Delta \varepsilon_{p}+\Delta \mu_{p}}{\varepsilon_{p}^{f}+\mu_{p}^{f}}$$

(5) $$\varepsilon_{p}^{f}+\mu_{p}^{f}=D_{1}{\left(P*+T*\right)}^{D_{2}}$$

where $\Delta \varepsilon_{p}$ and $\Delta \mu_{p}$ are the equivalent plastic strain increments and the plastic volumetric strain increments of the elements in a computational cycle. Here, $\varepsilon_{p}^{f}$ and $\mu_{p}^{f}$ are the equivalent plastic strain and plastic volumetric strain at an ordinary pressure, respectively. $T*=T/f_{c}{}^{\prime }$ is the maximum tensile pressure that the material can bear, and T is the tensile strength. $D_{1}$ and $D_{2}$ are the damage constants of the concrete material. When P*=−T*, the concrete can no longer bear any plastic strain. The parameter $\varepsilon_{f\min}$ is the amount of plastic strain before fracture, such that $D_{1}{\left(P*+T*\right)}^{D_{2}}\geq \varepsilon_{f\min}$. Figure 4b shows the damage model.

#### 3.1.3. Equation of State

The relationship between the hydrostatic pressure and the volumetric strain of the concrete is expressed by the segmental state equation shown in Figure 4c. Stage *OA* describes the linear elastic stage during which $p<p_{c}$, and both the loading section and unloading section can be expressed by:

(6) $$p=K_{e}\mu$$

where $K_{e}$ is the bulk modulus, $p_{c}$ and $\mu_{c}$ are the crushing pressure and crushing volumetric strain in the uniaxial compressive experiment, respectively.

Stage *AB* is a plastic transition stage during which $p_{c}\leq p<p_{l}$, and plastic deformation occurs as the voids of concrete are compressed.

Loading section:

(7) $$p=p_{l}+\frac{\left(p_{l}-p_{c}\right)\left(\mu -\mu_{l}\right)}{\mu_{l}-\mu_{c}}$$

where $p_{l}$ is the locking pressure, and $\mu_{l}$ is the locking volumetric strain.

Unloading section:

(8) $$p-p_{\max}=\left[\left(1-F\right)K_{e}+FK_{l}\right]\left(\mu -\mu_{\max}\right)$$

(9) $$F=\frac{\mu_{\max}-\mu_{c}}{\mu_{l}-\mu_{c}}$$

where $K_{l}$ is the plastic volumetric modulus, $p_{\max}$ and $\mu_{\max}$ are the maximum volumetric pressure and volumetric strain before unloading in which the holes of the concrete are expelled and damage occurs accompanied by cracks.

Stage *BC* is a fully compacted stage during which $p>p_{l}$, when the pressure reaches $p_{l}$, the holes are crushed completely. The relationship between p and μ is represented by a cubic polynomial.

Loading section:

(10) $$p=k_{1}\bar{\mu }+k_{2}{\bar{\mu }}^{2}+k_{3}{\bar{\mu }}^{3}$$

(11) $$\bar{\mu }=\frac{\mu -\mu_{l}}{1+\mu_{l}}$$

where $\bar{\mu }$ is the amended volumetric strain, and $k_{1}$, $k_{2}$ and $k_{3}$ are constants.

Unloading section:

(12) $$p-p_{\max}=K_{l}\left(\bar{\mu }-{\bar{\mu }}_{\max}\right)$$

There is no hole in this stage, and the concrete is crushed completely.

### 3.2. Modified Strength Yield Surface

The parameters representing the strength yield surface for concrete-like materials were mostly taken as the original parameters revealed by Holmquist et al. [24], which was acquired from concrete with a compressive strength of 48 MPa, which is not reasonable for RCC material. The strength parameters of RCC in the HJC model contain A, B, N and $\sigma {*}_{\max}$, which can be determined from the triaxial compression experimental data based on plastic yield surface theory [26]. 

**Figure 5:**
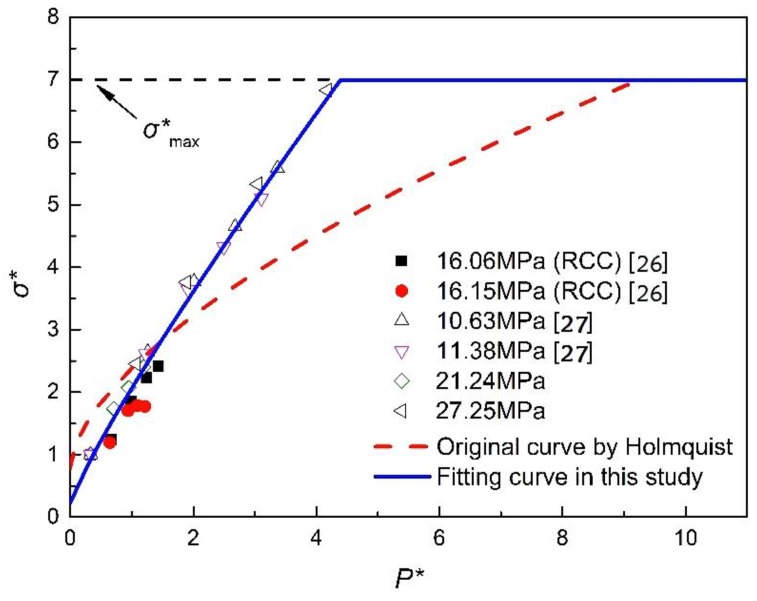
Determinations of the yield surface by triaxial compression data.

Based on the triaxial compressive experimental data derived from this study and data from previous studies [27,28,29], some parameters on the strength yield surface have been calibrated according to the strength theory of materials mechanical and curve fitting method. Figure 5 shows the yield surface ascertained by experimental data of RCC and normal concrete. It can be seen that A is significantly smaller than the original value of 0.79, which is coincident with the conclusion given by Malvar et al. [11]. Finally, the equation of the strength yield surface can be expressed as shown below, ignoring the effects of damage and strain rate:

(13) $$\sigma *=0.23+1.84P{*}^{0.88}$$

### 3.3. Modified Strain Rate Effect

**Figure 6:**
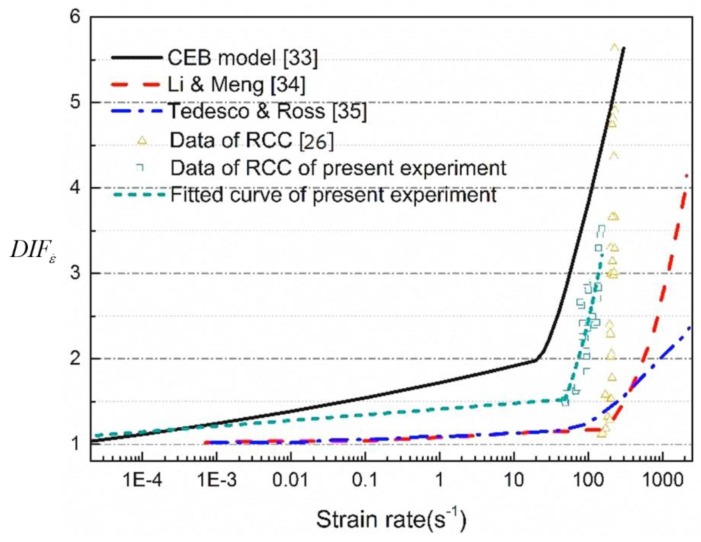
The true compressive strength $DIF_{\dot{\varepsilon }}$ of RCC material.

Many factors affect the compressive strengths directly obtained from the RCC experimental tests, and attention has been paid by researchers [23,30,31], especially on the dynamic strength increment, which is a combination of the strain rate effect and the lateral inertial confinement. Figure 6 shows the relationship between the $DIF_{\dot{\varepsilon }}$ (compressive dynamic increase factor) and the average strain rate of normal concrete from SHPB experimental results in various publications [32,33,34], and the data points in Figure 6 represent the experimental result of RCC whose time change law is similar to the CEB (Comite Euro-International du Beton) model. The findings are also consistent with those of other researchers [1,35]. The material strength enhancement using the DIF_TOT_, directly obtained from laboratory tests, will overestimate the true dynamic material strength. Therefore, the lateral inertia confinement effects need to be removed to derive the true dynamic material strength at high strain rates. In our previous study [1], numerical simulations were carried out to quantify the lateral inertia confinement effect on the concrete strength increment with respect to strain rate. The same approach is adopted here. After the lateral inertia confinement, the contribution to the strength increment is removed. The empirical formulas for the $DIF_{\dot{\varepsilon }}$ of RCC material in terms of compressive strength are derived below and will be adopted in the modified HJC model.

(14) $$DIF_{\dot{\varepsilon }}=\left\{ \begin{array}{cc} 1.2619+0.0524\log\dot{\varepsilon } & \dot{\varepsilon }<50s^{-1}\\ 3.4326-1.7692\log\dot{\varepsilon }+{0.3151\log}^{2}\dot{\varepsilon } & \dot{\varepsilon }\geq 50s^{-1} \end{array}\right.$$

### 3.4. Parameter Calibration for the Modified HJC Model

The basic parameters in the HJC model for RCC, which are ρ (density), G (shear modulus) and $f_{c}{}^{\prime }$ (quasi-static compressive strength), can be determined by the quasi-static experiment. The elastic modulus E=2G(1+ν) and the bulk modulus $K_{e}=E/3(1-2\nu )$ can also be obtained, and ν is Poisson’s ratio. The maximum tensile hydrostatic pressure T can be determined by the ACI equation [36] below:

(15) $$T=0.62\sqrt{f_{c}{}^{\prime }}$$

The equation of the state is mainly drawn from the Hugoniot curve, and the state of RCC would not reach the third stage, so the parameters $k_{1}$, $k_{2}$ and $k_{3}$ are taken as the original values. $p_{c}$ and $p_{l}$ can refer to the formula proposed by Holmquist et al. [24] as well. Other parameters are the crushing volumetric strain $\mu_{c}=p_{c}/K_{e}$, locking volumetric strain $\mu_{l}=\frac{\rho_{{}_{grain}}}{\rho_{0}}-1$, which can be easily obtained, and $\rho_{grain}$ which is the compacted density of concrete.

Holmquist et al. [24] assumed that the damage parameter is independent of the material strength, so $D_{1}$, $D_{2}$ and $\varepsilon_{f\min}$ are the same as the original parameters. For concrete-like material, crushing is the main failure form that can be described by fracture mechanics theory and applied to the simulation analyses. The stress–strain relationship and the cracks distributed in the concrete are considered, and different methods are employed to simulate the behaviour of the concrete under high strain rate loading [37,38]. The proper failure criterion should be included for describing the failure and erosion of concrete, and many kinds of failure criteria can be used to determine the status of the material; the maximum principal strain failure criterion and shear strain failure criterion are the most suitable factors for describing the failure status of concrete-like materials [39]. Therefore, combining the specimen failure process and morphology shot by a high-speed camera, the maximum principal strain failure criterion is adopted, which means that when $\varepsilon_{1}\geq \varepsilon_{\max}$, the element is in failure and deleted from the model and loses its carrying capacity, in which $\varepsilon_{1}$ is the maximum principal stress, and $\varepsilon_{\max}$ is the maximum principal stress at failure [40]. Table 3 lists the modified HJC parameters of RCC (C20). 

## 4. Numerical Verification

To evaluate the performance of the modified HJC model in predicting and reproducing the dynamic response of concrete structures under impact loading, the SHPB test of concrete is simulated using LS-DYNA finite element software, and the test results of the existing experimental tests on ∅50–25 mm concrete specimens with the SHPB test device are selected to validate the accuracy of the numerical simulation. 

### 4.1. Numerical Model

**Figure 7:**
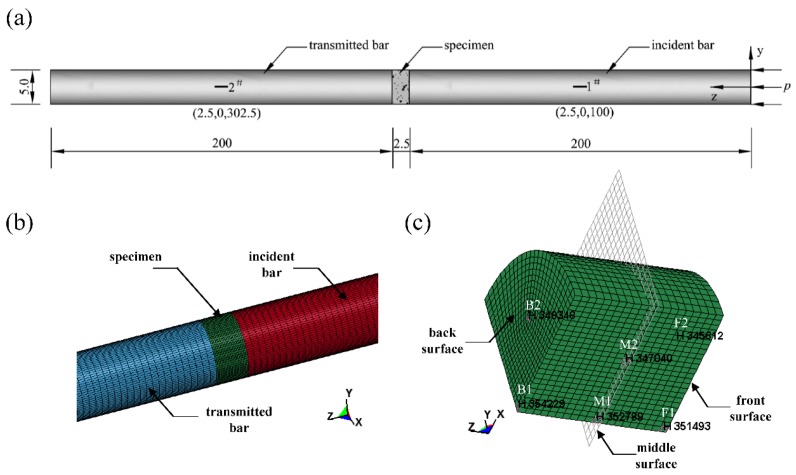
Numerical model of SHPB systems. (**a**) SHPB system and gage points at the bars (unit: cm). (**b**) Local model. (**c**) Specimen and gage points.

In the SHPB test simulation, the geometric model consists of four parts, i.e., the concrete specimen, the impact bar, the incident bar and the transmission bar are shown in Figure 7. Considering the symmetry of the SHPB system, a quarter of the simulation specimen is modelled to significantly reduce the model size and computation time. The mesh size effect of shock wave peak pressure at different scaled distances were analysed and compared and a convergence test was carried out to determine the mesh size. The incident bar and transmission bar are both 2.0 m long, and they are dispersed to meshes of 0.005 m. The specimen is dispersed to meshes of 0.00125 m according to our previous work [41] using “explosive radius and the mesh size ratio” as a criterion for determining mesh size. The non-reflecting boundary condition is applied for the two sides of the model that are perpendicular to each other. The eroding surface is applied to describe the contacting characteristics between the pressure bars/specimen surfaces, and the physical crushing and fracture can be captured well [42]. The pressure bars, which are made of high strength steel springs, are assumed to be elastic with the main parameters as follows: the density is equal to 7800 kg·m^3^, the Young’s modulus is equal to 200 GPa and the Poisson’s ratio is 0.25. The RCC specimen is simulated by the modified HJC model with parameters determined in Section 3. Two monitoring units are set at the surface of the bar centre (1^#^ is at the incident bar and 2^#^ is at the transmission bar) to gather data, and the location of gage points 1^#^ and 2^#^ is suitable for acquiring the stress–strain relationship of the specimen. F1 and F2 are the gage points on the front surface of the specimen, M1 and M2 are the gage points on the middle surface of the specimen, and B1 and B2 are the gage points on the back surface of the specimen.

### 4.2. Stress–Strain Curve

**Figure 8:**
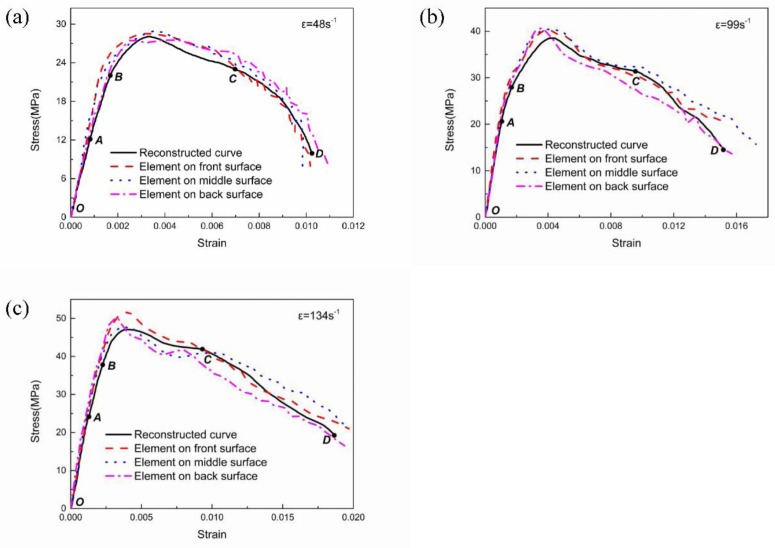
Stress–strain curves with different strain rates. (**a**) stress–strain curves with strain rate of 48 s^−1^, (**b**) stress–strain curves with strain rate of 99 s^−1^, (**c**) stress–strain curves with strain rate of 134 s^−1^.

The stress–strain relationship of the stress/strain monitoring elements in specimens and three-wave method results calculated by the simulation are compared in Figure 8. Using a custom-shaped punch can achieve a loading with a single frequency half-sine wave to the pressure bar and according to the strain wave measured during the SHPB experiment, a half-sine wave with peak values of 75, 125, 175 and 225 MPa is applied severally on the front surface of the incident pressure bar and is the boundary input in the simulation. Figure 8a–c shows the stress–strain curves under different strain rates, which correspond to the SHPB experiment. Taking the stress history and strain history of the three elements at the front surface, middle surface and back surface of the specimen (elements should avoid nearing the specimen edge) eliminates the time to acquire the stress–strain curves as the dashed lines show. The full line represents the stress–strain relationship obtained by the three-wave method. The results with three strain rates of, 48, 99, and 134 s^−1^, which correspond to the loading stress amplitudes of 75, 125 and 175 MPa, are shown. Before the maximum stress is achieved, the curve of the typical elements and the reconstructed curve agree well. After the damage begins to develop, there is a certain degree of discretisation. For the typical elements, the descent stage of the curve is not smooth because of the evolution of the crushing damage, and the higher the loading strain rate is, the more discrete the stress–strain curves are.

The stress–strain curve can be divided into four sections: the linear elastic ascending stage (OA), the initial damage stage (AB), the severe damage evolution stage (BC) and the structural failure stage (CD). The stress–strain relationships of the reconstructed element and the typical elements are well fitted. Differences among the elements on the three surfaces appear when the specimen is at the initial damage stage, which is caused by the stress wave propagation and its reflection and refraction in the specimen, and this discrepancy deviates from the reconstructed curve. The SHPB experiment reflects the average behaviour of the specimen, which reduces the effect of the heterogeneity of the concrete material, so the existence of a low level of stress heterogeneity, as mentioned above, is allowed [43]. Before the stress reaches the peak value, damage occurs in some parts of the specimen. The discreteness of the stress–strain curves reflects the difference in the damage evolution caused by the transverse Poisson effect intensifying the multidimensional stress state, and the oscillation in the later stage is mainly brought out by the element deletion. The average stress value of the three elements is larger than the reconstructed stress, mainly because the typical element is located on the specimen centre, which is not severely damaged. Above all, using a 50 mm-diameter SHPB apparatus with a custom-shaped punch that can produce a half-sine strain wave and without consideration of the defect of the specimen, the stress uniformity state can be reached, and the valid range of the stress–strain curve is from the linear elastic ascending stage to the severe evolution of the damage stage. In the last stage, cracks pass through the whole specimen, and the specimen severely deforms and fragments. The stress–strain curve at this stage not only represents the behaviour of the concrete material but also reflects the structural characteristics.

**Figure 9:**
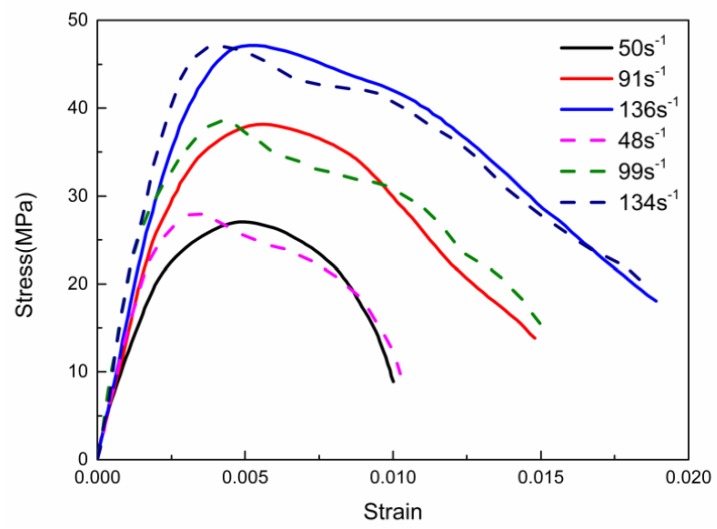
Stress–strain curves of the experiment and simulation with solid lines depicting the experimental curves and dashed lines representing numerical curves.

Figure 9 shows the stress–strain curves under different strain rates obtained from the SHPB experiment, whose average strain rates are 50, 91 and 136 s^−1^, respectively, corresponding to the amplitudes of the loading pressures of 75, 125 and 175 MPa in the simulation. The solid lines depict the result from the SHPB experiment, while the dashed lines represent the stress–strain relationship calculated by the three-wave method, and the mechanical behaviour shown by the curves is consistent with the analysis above. It can be seen that the strain rate strengthening effect increased with the enhancement of the loading strain rate, and the arc curvature during the severe evolution of the damage stage decreased with the strain rate increment, which indicates a greater adequacy of the damage evolution. Comparing the stress–strain relationships of the RCC specimen between the SHPB experimental result and the numerical simulation via loading with a half-sine pressure wave with different amplitudes on the incident bar, it can be proved that using the parameters of the modified HJC model determined above to describe the dynamic compressive mechanical behaviour of the RCC specimen is effective and feasible.

### 4.3. Failure Criterion and the Damage Mode

**Figure 10:**
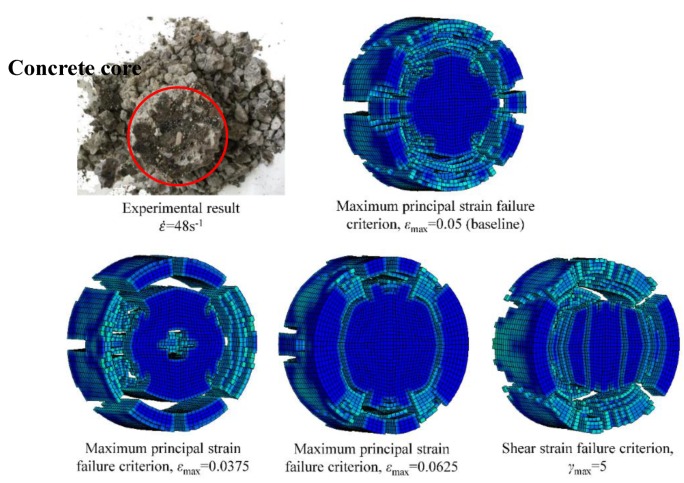
Comparative failure patterns of the specimens after being impacted with the test results.

The failure criterion is crucial to the failure mode and other responses of the structure subjected to the impact and explosion loadings. The influence of the failure criterion in the modified HJC model on the specimen failure mode is shown in Figure 10, and the simulated result is compared with the experimental result. The erosion failure criterion of MAT_ADD_EROSION is introduced to control the failure of the elements, which mainly affects the unloading section. The experimental result shows that the specimen retains a core and that the failure develops around the surrounding area. Adopting the maximum principal strain failure criterion can better depict the specimen failure than the shear strain failure criterion, and $\varepsilon_{\max}=0.05$ is recommended for RCC due to a better simulation results for the experimental failure pattern illustrated in Figure 10.

**Figure 11:**
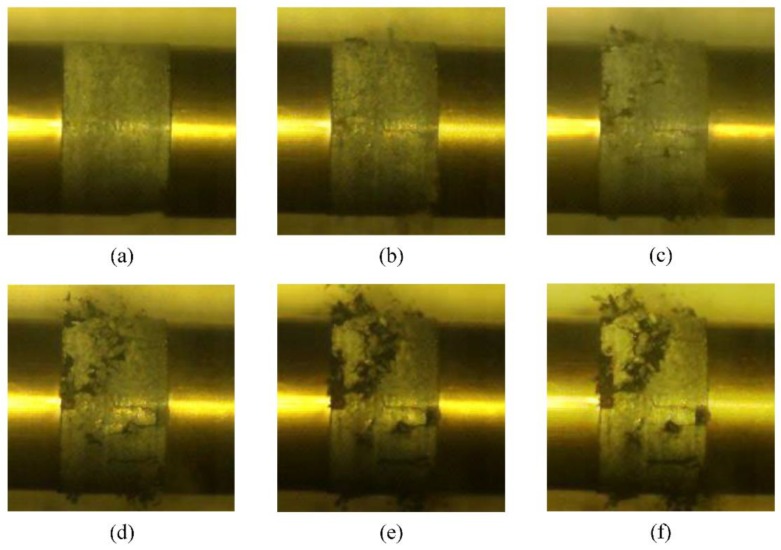
Failure process of a 50 mm diameter specimen. (**a**) about 308 μs, (**b**) about 498 μs, (**c**) s about 698 μs. (**d**) about 898 μs, (**e**) about 1098 μs, (**f**) about 1298 μs.

**Figure 12:**
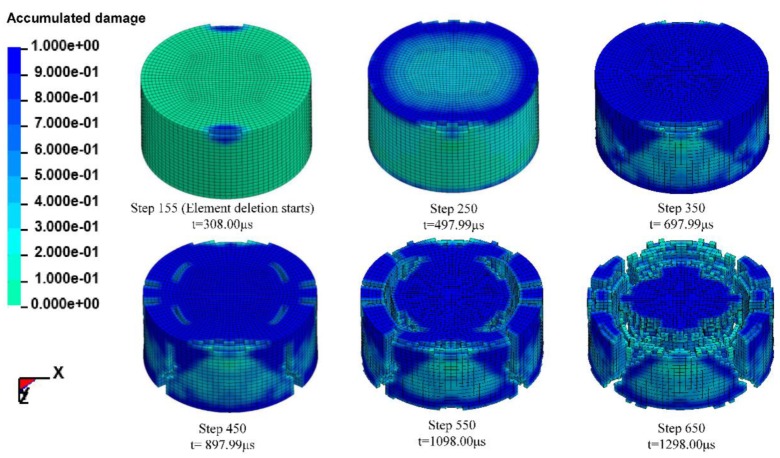
RCC specimen failure process and the accumulated damage.

Figure 11 shows the failure process of the RCC specimen captured by a high-speed camera during the SHPB experiment. Cracking occurs in the specimen surface immediately after the stress wave is applied. Then, the specimen is damaged near the end of the transmission bar, which further spreads to the entire specimen and scatters. Finally, the specimen is destroyed and loses its load carrying capacity. Figure 12 shows the simulated failure processes of the RCC specimens under nearly the same average strain rate of 48 s^−1^. The failure develops slowly, and the damage degree is low, which first occurs at the edge of the front surface and back surface. Then, as the stress wave spreads along the *z*-axis direction, the damage grows continuously, and for the reflection and the refraction of the stress, an increasing number of elements fail until the cracks run through the whole specimen. For a relatively low loading strain rate, the specimen damage pattern exhibits the phenomenon of core retention. This damage process coincides with the pattern captured by the high-speed camera, proving that using the numerical simulation method to reproduce the indoor SHPB experiment for qualitative analysis is feasible.

**Figure 13:**
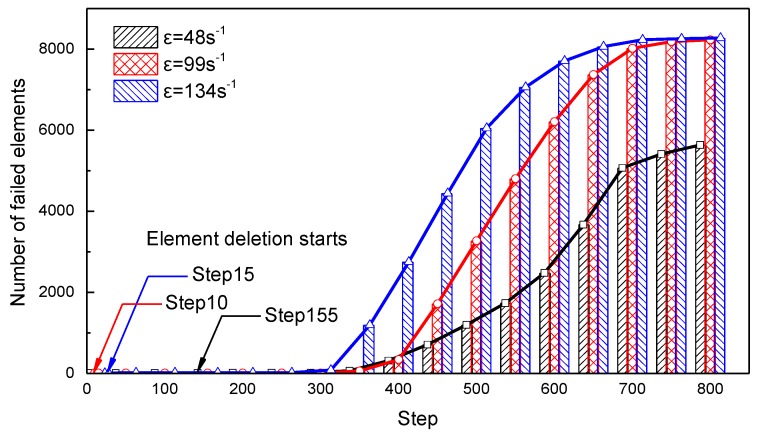
Failure element trend correlation during the simulation.

Figure 13 shows the accumulated amount of failed elements of the specimen, which is scattered to 8640 elements under three loading strain rates. It is shown that at the strain rates of 48, 99 and 134 s^−1^, element deletion starts at step 15, step 10 and step 155, respectively, and more elements fail at higher strain rates. The loading stress wave arrives at the specimen at approximately 350 μs (step 150), but the element failure is delayed, which is obvious at 800 μs (step 200). After 400 μs, the number of failed elements grows sharply, especially at the strain rate of 99 and 134 s^1^, until most of the elements fail, and the specimen loses its load supporting capability.

## 5. Parameter Studies

As is known, there are many parameters, including concrete’s compressive and tensile strengths, modulus of elasticity and density, etc., that may influence on the numerical simulations. To illustrate the effect degree and differences between modified strength yield surface and the original one, four parameters (A, B, N and C) in the yield function (Equation (3)) have been selected to make numerical simulations of RCC under impact loading. In this section, the sensitivity of these parameters to the modified HJC model is analysed by varying one parameter by ± 25 percent of the value in Table 3 while the others are kept as the baseline. The loading stress wave has a peak value of 175 MPa, and the computational model is the same as that described in Section 4.1.

**Figure 14:**
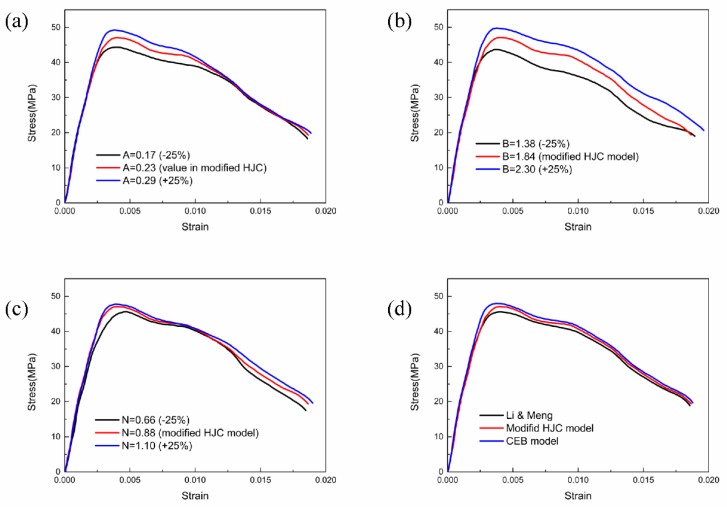
Effect of strength parameters on the stress–strain curve: (**a**) effect of parameter A on the stress–strain curve, (**b**) effect of parameter B on the stress–strain curve, (**c**) effect of parameter N on the stress–strain curve, (**d**) effect of parameter C on the stress–strain curve.

The influences of the normalized parameters on the compressive properties are shown in Figure 14a,b comparing the reconstructed stress–strain curves. The peak value of stress increases with the increasing increment of A while the peak strain remains unchanged; the rising step of the stress–strain curve becomes steeper, and the descending stage becomes slower. A is the normalized cohesive strength in the yield equation, and a smaller A corresponds to a reduction in the cohesive strength in terms of a decrease in the peak stress. When considering the damage, A is the coefficient of the damage term that controls the proportion of the damage in the yield surface, and a smaller A indicates a less apparent influence on the yield surface, so the descending stage of the stress–strain curve is slower. Figure 14b shows that the variation in B has no effect on the elastic stage of the stress–strain curve; however, the peak stress increases with the increasing increment of B, and the gradient of the yield increase stage becomes larger. The peak value of strain and the slope of the descending segment are insensitive to the value of B. B controls the proportion of the pressure in the yield equation, and its variety only affects the peak stress, but the overall shape of the curve is invariant. Although A and B share similar controlling effects on the peak value of the stress, their physical meanings are distinct.

Figure 14c shows the stress–strain curves with different exponents of the normalized hydrostatic pressure, which has an obvious influence on the gradient of the yield ascending curve. With the increment of N, the yield ascending stage becomes slower, and the width of the curve becomes narrower, indicating a process of softening. A reasonable and accurate value of N will determine the shape of the stress–strain curve.

Figure 14d presents the reconstructed stress–strain curves with different strain rate effects. The CEB model considers the dynamic increment, which contains the strain rate effect and the lateral confinement effect, so the stress–strain wave under this condition has the largest peak stress. Li et al. [32] eliminated the influence of the lateral confinement effect as well as the modified HJC model in the present study, resulting in lower strength increments, but the strain rate effect on RCC is more obvious than that on normal concrete. Adopting different formulas for the dynamic increment factor that governs the strain rate influence factor in the yield equation will change the magnitude of the peak stress but has a trivial influence on the overall shape of the stress–strain curve.

## 6. Conclusions

Based on the original HJC model and combining the SHPB experimental data and the triaxial compressive test results of RCC, a modified constitutive model is developed to describe the dynamic compressive properties of RCC. The main contributions and conclusions can be drawn as follows:

(1) The triaxial compressive test and SHPB experimental results of RCC are provided as the basis of the modified HJC model. Then, the strength yield surface and the strain rate effect represented in the HJC model are modified by combining the triaxial compressive test results of concrete (including RCC and normal concrete for the limitation of the effective experimental data) and the SHPB experimental data of RCC, while the maximum principal strain failure criterion is adopted to describe the crushing and fracture properties of RCC. Parameters in the modified HJC model are obviously different from the values in the original model, and these corrections contribute to a more credible and accurate constitutive model to predict the dynamic mechanical characteristics of RCC.
(2) The improved simulation method is established by the parameters obtained from the modified HJC model. The custom-shaped punch is replaced by loading a half-sine stress wave at the front surface of the incident bar, and contact of the eroding surface to the surface is employed, which shows great consistency with the SHPB experiment.
(3) An improvement in the simulation with the parameters obtained from the modified HJC model is demonstrated compared to the test data. Stress–strain curves from the SHPB simulation results agree well with the experimental results, and the typical element at different parts of the specimen shows a similar stress–strain law to that of the results from the three-wave method.
(4) The influence of the failure criterion, normalized parameters, pressure hardening exponent and strain rate effect on the dynamic compressive characteristics of RCC are analysed. The parameters referred to on the yield surface mainly affect the ascending section of the stress–strain curve, while the failure criterion basically controls the unloading section. Adopting the maximum principal strain failure criterion can better depict the specimen failure than the shear strain failure criterion, and $\varepsilon_{\max}=0.05$ is recommended for RCC so that the failure process of the RCC specimen simulated via the modified HJC model is consistent with the results captured by the high-speed camera. The results of the dynamic mechanical behaviour of RCC specimens obtained from the SHPB experiment and simulation are reliable and effective and agree well with each other.

## Figures and Tables

**Table 1:** Mixture and material properties of Roller Compacted Concrete (RCC).

W/C	Sand Ratio (%)	Fly Ash Content (%)	Water Reducing Agent (%)	Air Entraining Agent (%)	Material Consumption (kg/m^3^)					Air Content (%)	Wet Density (kg/m^3^)
					Water	Cement	Fly Ash	Sand	Aggregate		
0.50	31	60	0.8	0.05	88	70	106	672	1507	3.8	2453

Note: W/C: water to cement ratio

**Table 2:** Triaxial compression results of RCC.

$\sigma_{3}(\mathrm{MPa})$	$\sigma_{3}/f_{c}^{\prime }$	Peak Additional Axial Stress $\Delta \sigma_{p}(\mathrm{MPa})$	Peak Axial Strain $\varepsilon_{1p}/\%$	Peak Lateral Strain $\varepsilon_{3p}/\%$
0	0	14.9	0.411	−0.237
5	0.34	26.1	0.625	−0.490
10	0.67	37.3	1.578	−0.760
15	1.01	41.9	2.270	−1.279
20	1.34	50.3	3.078	−2.228
25	1.68	54.1	3.630	−2.849
30	2.01	60.1	4.516	−3.478

**Table 3:** HJC model parameters for RCC (C20).

Fundamental Parameters		Yield Surface		Damage Parameters		EOS Parameters	
ρ	2350 kg·m^−3^	A	0.23	$D_{1}$	0.04	$p_{c}$	7.0 MPa
G	10.63 GPa	B	1.84	$D_{2}$	1.00	$\mu_{c}$	0.005
$f_{c}{}^{\prime }$	20.68 MPa	N	0.88	$e_{f\min}$	0.01	$p_{l}$	800 MPa
T	2.80 MPa	$\sigma {*}_{\max}$	7.00			$\mu_{l}$	0.12
						$k_{1}$	85 GPa
						$k_{2}$	−171 GPa
						$k_{3}$	208 GPa
